# Supplementary material for: Content-rich biological network constructed by mining PubMed abstracts
Source: BMC Bioinformatics. 2004 Oct 8;5:147. doi: 10.1186/1471-2105-5-147 (PMC528731; doi:10.1186/1471-2105-5-147)
Supplement: Additional File 2 — The original results of the above study (non-essential files are deleted to keep the file size under the limit set by BMC bioinformatics). [file 1471-2105-5-147-S2.bz2 › chilibotAdditionalFile2/dip50/504ID10880444E10671/html/BMP2_BMPR2.html]

 


 **BMP2** and **BMPR2** 
  
Found 51 abstracts in PubMed, retrieved 50.  
 

 What does Google say? 
 PDF only 
| .edu only 

---

**Interactive relationship** (e.g. stimulation, inhibition, etc)

**Neutral relationship**- A mammalian serine threonine kinase  [ **BMPR2** ]  receptor specifically binds BMP 2  [ **BMP2** ]  and BMP 4.  Ref: 7811286 Biochem Biophys Res Commun, 1994
- To better understand the roles of BMPs in Xenopus laevis embryogenesis, we have cloned a cDNA coding for a serine threonine kinase  [ **BMPR2** ]  receptor that binds BMP 2  [ **BMP2** ]  and BMP 4.  Ref: 7937936 Proc Natl Acad Sci U S A, 1994
- BMPR II  [ **BMPR2** ]  is a transmembrane serine threonine kinase  [ **BMPR2** ]  that binds BMP 2  [ **BMP2** ]  and BMP 7 in association with multiple type I receptors, including BMPR IA Brk1, BMPR IB, and ActR I, which is also an activin type I receptor.  Ref: 7791754 Mol Cell Biol, 1995
- Requirement of BMP 2  [ **BMP2** ]  induced phosphatidylinositol 3 kinase and Akt serine threonine kinase  [ **BMPR2** ]  in osteoblast differentiation and Smad dependent BMP 2  [ **BMP2** ]  gene transcription.  Ref: 12084724 J Biol Chem, 2002
- The binding epitopes of BMP 2  [ **BMP2** ]  for BMPR IA type I and BMPR II  [ **BMPR2** ]  or ActR II type II were characterized using BMP 2  [ **BMP2** ]  mutant proteins for analysis of interactions with receptor ectodomains.  Ref: 10880444 EMBO J, 2000
- BMP 2  [ **BMP2** ]  antagonists emerge from alterations in the low affinity binding epitope for receptor BMPR II  [ **BMPR2** ] .  Ref: 10880444 EMBO J, 2000
- To determine the molecular basis for these effects, we demonstrated that BMP 2  [ **BMP2** ]  is bound to mIMCD 3 cells by the type I serine threonine kinase  [ **BMPR2** ]  receptor, ALK 3,.  Ref: 9435686 Am J Physiol, 1997
- TGF beta related factors, including BMP 2  [ **BMP2** ]  4, Dpp and activin, act through two types of serine threonine kinase  [ **BMPR2** ]  receptors which can form a heteromeric complex.  Ref: 8774881 Nature, 1996
- BMP 2  [ **BMP2** ]  and BMP 7 can induce a transcriptional response when added to cells coexpressing ActR I and BMPR II  [ **BMPR2** ]  but not to cells expressing either receptor alone.  Ref: 7791754 Mol Cell Biol, 1995
- BRK 3  [ **BMPR2** ]  is a vertebrate type II receptor for BMP 4 distantly related to invertebrate type II receptors for BMP 2  [ **BMP2** ]  BMP 4 dpp, such as daf 4 and punt.  Ref: 7488098 Biochem Biophys Res Commun, 1995
- METHODS To identify amino acid side chains involved in receptor binding, a collection of in vitro mutagenized human BMP 2  [ **BMP2** ]  variants was prepared and subjected to interaction analyses with use of the receptor ectodomains of BMPR IA, BMPR II  [ **BMPR2** ] , and ActR II immobilized on a biosensor system.  Ref: 11263668 J Bone Joint Surg Am, 2001
- Additionally, immunostaining for BMP 2  [ **BMP2** ]  4 and OP 1 was observed in multinucleated osteoclast like cells on the newly formed trabecular bone, along with BMPR II  [ **BMPR2** ] .  Ref: 9626398 Bone, 1998
- BMP 2  [ **BMP2** ]  stimulated Akt serine threonine kinase  [ **BMPR2** ]  activity in a PI 3 kinase dependent manner in osteoblast precursor cells.  Ref: 12084724 J Biol Chem, 2002
- BMPR II  [ **BMPR2** ]  binds BMP 2  [ **BMP2** ]  and 7 on its own, but binding is enhanced by coexpression of type I BMP receptors.  Ref: 7791754 Mol Cell Biol, 1995
- In the early stage of fracture repair, the expression of BMP 2  [ **BMP2** ]  4 and OP 1 was strongly induced in the thickened periosteum near the fracture ends, and coincided with an enhanced expression of BMPR II  [ **BMPR2** ] .  Ref: 9626398 Bone, 1998

**Non-interactive relationship** (e.g. studied together, co-existance, homology, etc.)

- **BMP2**  and BMP4 are produced by the thymic stroma and the requisite BMP receptor molecules BMPR 1A, BMPR 1B, BMPR II  [ **BMPR2** ] , and signal transduction molecules Smad, 8, and 4 are expressed by DN thymocytes.  Ref: 12421925 J Immunol, 2002
- With use of immunohistochemical analysis, we studied the localization of BMP 2  [ **BMP2** ] , BMP 4, and BMP 7 and their receptors BMPR IA, BMPR IB, and BMPR II  [ **BMPR2** ]  as well as pSmad1.  Ref: 12429748 J Bone Joint Surg Am, 2002
- Addition of the BMPR II  [ **BMPR2** ]  specific ligands,  **BMP2**  and BMP4, in the culture medium of the endothelial cells seeded on type I collagen increased 3 H thymidine incorporation into cellular DNA, indicating that endothelial cells were able to form a functional receptor.  Ref: 11327700 Biochem Biophys Res Commun, 2001
- The purpose of this study was to determine the locations of BMP 2  [ **BMP2** ]  4, osteogenic protein 1 OP 1, also termed BMP 7, and BMP type II receptor  [ **BMPR2** ]  BMPR II  [ **BMPR2** ]  during rat fracture healing by immunostaining, and thereby elucidate the possible roles of the BMPs and BMPR II  [ **BMPR2** ]  in intramembranous ossification and endochondral ossification.  Ref: 9626398 Bone, 1998
- 0.01 increase of BMP 2  [ **BMP2** ] , BMP receptor R IA, and BMPR II  [ **BMPR2** ]  messenger RNA levels, respectively.  Ref: 10220513 Gastroenterology, 1999
- In this study, we studied immunohistochemically the spatial and temporal localization of BMP 2  [ **BMP2** ]  4, osteogenic protein 1 OP 1, or BMP 7, and BMP receptors BMPR IA, BMPR IB, and BMPR II  [ **BMPR2** ]  in the epiphyseal plate of growing rats.  Ref: 9703876 Anticancer Res< MedlineTA>Anticancer Res, 1999
- BMP 2  [ **BMP2** ]  signals via oligomerization of type I and type II serine threonine kinase  [ **BMPR2** ]  receptors.  Ref: 10692589 FEBS Lett, 2000
- RESULTS Transcription of BMP 2  [ **BMP2** ] , BMP 3, BMP 4, BMP 5, and BMP 7 and receptors of BMPR IA, BMPR IB and BMPR II  [ **BMPR2** ]  was detected in ex vivo and cultured epithelium and stroma.  Ref: 9950587 Invest Ophthalmol Vis Sci, 1999
- Immunohistochemical analysis demonstrated that cells expressing either BMP 2  [ **BMP2** ]  4 or their receptors BMPR IA, BMPR IB, and BMPR II  [ **BMPR2** ]  preferentially appeared in nodules.  Ref: 9600781 Bone, 1998
